# Usefulness and Limits of DOAC Removal Agents Based on Activated Charcoal in Thrombophilia Testing: Literature Review and Expert Proposals

**DOI:** 10.1055/a-2695-2674

**Published:** 2025-09-29

**Authors:** Georges Jourdi, Claire Flaujac, Emmanuel De Maistre, Nicolas Gendron, Valérie Eschwège, Laetitia Mauge, on behalf of the French Society on Thrombosis Haemostasis

**Affiliations:** 1 Service d'Hématologie Biologique, Hôpital Lariboisière, Assistance Publique Hôpitaux de Paris Nord (APHP.Nord), Paris, France; 2 Université Paris Cité, INSERM, UMRS-1144, Optimisation Thérapeutique en Neuropharmacologie, Paris, France; 3 Laboratoire de Biologie Médicale (Secteur Hémostase), Centre Hospitalier de Versailles - Hôpital André Mignot, Le Chesnay-Rocquencourt, France; 4 Laboratoire d'Hémostase, CHU Dijon Bourgogne, Dijon, France; 5 Service d'Hématologie Biologique, Centre-Université de Paris (APHP.CUP), Hôpital Européen Georges Pompidou, Assistance Publique Hôpitaux de Paris, Paris, France; 6 Paris Cardiovascular Research Center, Team Endotheliopathy and Hemostasis Disorders, Université Paris Cité, INSERM, Paris, France; 7 F-CRIN, INNOVTE, Saint-Etienne-France; 8 Service d'Hématologie Biologique, Centre Hospitalier Universitaire de Nancy, France

**Keywords:** direct oral anticoagulant, lupus anticoagulant, antithrombin, protein C, protein S

## Abstract

Although inherited and acquired thrombophilia screening should ideally be performed outside of any direct oral anticoagulant (DOAC) therapy, it is sometimes performed in patients who are anticoagulated. However, DOACs have been shown to interfere with many hemostasis tests, with a risk of false-positive/negative results in lupus anticoagulant testing and overestimation of natural coagulation inhibitor levels, which may lead to misdiagnosis. Devices have been developed to overcome DOAC interference but their role in thrombophilia testing is not clearly established. In this comprehensive review, we provide an in-depth overview of the literature on the impact of DOACs on thrombophilia assays, including lupus anticoagulant testing, antithrombin, protein C, and protein S assessment. DOACs can interfere with the results of thrombophilia testing even at low concentrations; therefore information on current or recently discontinued anticoagulant treatment should be provided when prescribing thrombophilia testing. Data on the usefulness of the most used DOAC removal systems based on activated charcoal to circumvent DOAC interference are heterogeneous. They are summarized in this critical review. Although activated charcoal could be useful to remove DOACs from plasma prior to thrombophilia testing, it may not be completely effective, particularly with apixaban. Hence, and in the light of the available literature, we provide 22 practical proposals for reliable thrombophilia testing and accurate result interpretation in samples from patients receiving DOACs and treated in vitro with activated charcoal.

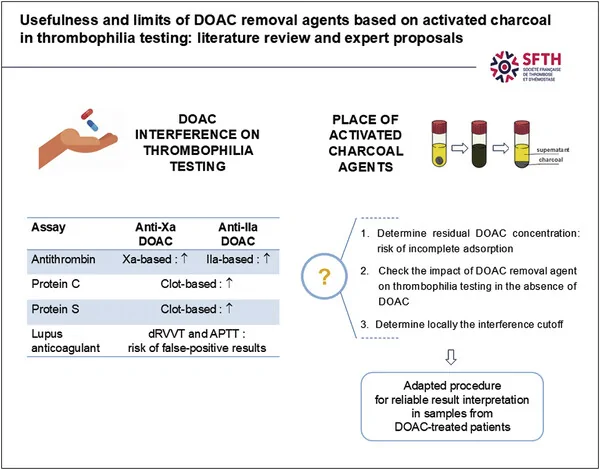

## Introduction

Venous thromboembolism (VTE) is a multifactorial disease resulting from the interaction among environmental, clinical, and biological risk factors. Thrombophilia testing is requested in select patients to identify those at high risk for recurrent VTE who may require indefinite anticoagulant therapy. 1 2 3 4 Thrombophilia testing typically includes screening for inherited thrombophilia, 5 such as antithrombin (AT), protein C (PC), and protein S (PS) deficiencies, as well as genetic testing for the c.1601G > A; p.Arg534Gln (also known as R506Q or factor V Leiden mutation—FVL) variant of *F5* gene and the c.*97G > A (prothrombin G20210A) variant of *F2* gene. In addition, thrombophilia testing includes the search for acquired biological risk factors such as antiphospholipid antibodies (aPL) detected by coagulation assays (lupus anticoagulant, LA) and immunological assays for anticardiolipin (aCL) and anti-β-2-glycoprotein I (anti-β2GPI) antibodies. 6 The risk of first VTE event or recurrent VTE depends on the type of thrombophilia and has been extensively detailed elsewhere. 7 8 9

Direct oral anticoagulants (DOACs) are now widely used for the treatment and prevention of VTE. 10 They include direct, specific, and reversible inhibitors of thrombin (dabigatran) or activated factor X (FXa) (apixaban, rivaroxaban, and edoxaban, i.e., oral FXa inhibitors). These oral drugs have predictable pharmacodynamic/pharmacokinetic profiles, do not require routine monitoring, and have similar or better efficacy and safety than traditional anticoagulants (including vitamin K antagonists [VKA] and heparin derivatives). 11 12 Hence, they are increasingly prescribed in clinical practice. Yet, it is well established that DOACs interfere with many of them, including inherited and acquired thrombophilia testing. 7 13 Although these assays should ideally be deferred until anticoagulation has been paused or discontinued for this purpose, 1 4 they are sometimes performed in patients who are still receiving DOACs. Indeed, thrombophilia testing can sometimes be prescribed by physicians who are not aware of the potential interference of DOACs with some thrombophilia testing. Alternatively, when inherited thrombophilia or antiphospholipid syndrome is highly suspected, physicians could decide to wait for thrombophilia testing results before stopping the treatment, as discontinuation could expose patients to a risk of thromboembolic recurrence. Besides, stopping DOAC treatment for 2 to 3 days prior to blood sampling for thrombophilia testing is not always sufficient to cancel the interference. The time required for complete DOACs elimination from the circulation varies from individual to individual perform thrombophilia testing before the introduction of anticoagulation 15 and residual DOAC concentrations that may still induce diagnostic interference depend on the DOAC and the assay used. 7 13 Another solution could be the use of DOAC-insensitive thrombophilia assays, but some inherited deficiencies could be missed with these assays and those developed for LA testing are neither widely available nor standardized. 14 However, such agents should specifically and completely remove DOAC compounds from plasma samples while being strictly neutral with regard to other plasma and reagent components.

On behalf of the French Society on Thrombosis and Haemostasis (SFTH), we analyzed and summarized the currently available data on DOAC interference with thrombophilia testing and, on the usefulness and limits of DOAC removal agents based on activated charcoal, and we provided guidance regarding reliable thrombophilia testing strategies in patients receiving DOACs.

## Methodology

A literature search was conducted in PubMed from inception to February 2025 using the following Medical Subject Headings (MeSH): (antithrombin OR protein C OR protein S OR lupus anticoagulant OR activated protein C resistance OR thrombophilia) AND (dabigatran OR rivaroxaban OR apixaban OR edoxaban) to report the interference of DOAC on thrombophilia testing. A second search based on (dabigatran OR rivaroxaban OR apixaban OR edoxaban) AND (activated charcoal OR DOAC Stop OR DOAC Remove) was performed to document the efficacy of activated charcoal agents to remove DOAC. Articles were then screened based on titles and abstracts to select appropriate ones for full-text review. The references of the selected articles were also searched for other potentially relevant studies. Therefore, analyzed data were from full-text available original articles reporting results on DOACs interference with thrombophilia testing and on the impact of DOAC removal agents on the assays, either in the absence or the presence of DOACs, in spiked or patient plasma samples.

## Results

### DOACs Interference with Thrombophilia Testing

#### Impact of DOACs on Inherited Thrombophilia Screening

Screening for natural anticoagulant deficiencies is partly based on the measurement of coagulation inhibitory activity, which can be overestimated in DOAC-containing samples, thus potentially masking true deficiencies. 7 Detection of FVL was initially based on plasmatic test searching for activated PC resistance (APCR). Due to its lack of sensitivity, this test is not recommended as a first-line assay in France, except in the rare cases of liver or bone marrow transplantation. 16 Genetic testing is instead recommended as a first-line assay to diagnose FVL mutation. 16 Detection of the FVL and *F2* c.*97G > A variants based on genomic DNA analysis is not affected by any anticoagulant in patient sample.

##### Antithrombin

Antithrombin is a serine protease inhibitor (serpin) that inhibits the activity of all serine proteases involved in the clotting process, mainly FIIa and FXa. Screening for AT deficiency (ATD) is based on the measurement of AT heparin cofactor activity (AT Cof) using chromogenic assays in the presence of either FIIa or FXa as AT target and heparin. 17 It allows the detection of all types of ATD (i.e., both quantitative and qualitative). In case of abnormal AT Cof result, the AT antigen level can be measured using immunological assays to differentiate between quantitative and qualitative ATD. DOAC targeting FIIa (dabigatran) may interfere with FIIa-based chromogenic assays, whereas oral FXa inhibitors may do so with FXa-based assays. 7 By increasing the degree of inhibition of FXa or FIIa, DOACs may lead to an overestimation of AT activity, increasing the risk of missing an ATD. This was first described in DOAC-spiked samples. These studies showed that DOAC interference is dose dependent and varies depending on the DOAC compound and the reagent used ( Table 1 ). 18 19 20 21 22 23 24 25 26 27 28 29 30 31 32 33 34 35 36 Vercruyssen et al confirmed the influence of oral FXa inhibitors on AT activity measurement based on FXa inhibition in deficiency-induced AT plasmas (by diluting normal pooled plasma from 6 healthy volunteers). 32 Based on in vitro studies, dabigatran interferes with AT activity measured using several FIIa-based assays only and in a concentration-dependent manner, with an overestimation of around 10% at plasma levels higher than 100 ng/mL. 19 20 21 22 33 Similarly, oral FXa inhibitors interfere with FXa-based assays in a concentration-dependent manner only at plasma concentrations above 100 ng/mL with all three drugs. 24 25 26 28 34 37 This was also observed in an ex vivo study where AT Cof was measured before and after rivaroxaban intake. 38 An increase in AT activity level of approximately 10 IU/dL per 100 ng/mL of oral FXa inhibitors has been reported. However, DOAC impact may vary depending on the DOAC compound. As an example, Gosselin et al observed that edoxaban interfered at concentrations higher than for rivaroxaban or apixaban on Hemosil Liquid AT reagent: from 269 ng/mL versus 130 ng/mL and 112 ng/mL respectively. 26 This study also illustrates the variable sensitivity of FXa-based assays to oral FXa inhibitors. For instance, the Innovance AT (Siemens) assay seems to be more affected than the Hemosil Liquid AT assay (Instrument Laboratory), with a minimal interfering concentration of 38 ng/mL of apixaban for Innovance AT versus 112 ng/mL for Hemosil Liquid AT. 26 This higher sensitivity of Innovance AT to DOACs was also observed with edoxaban.

**Table 1:** DOAC interference with antithrombin cofactor activity testing

Methods	Assay used	DOAC	DOAC concentration Range/Median [IQR]	DOAC removal agent	Impact of DOAC on AT result Minimal C% without impact	Reference
**FXa based assays**	***Coamatic LR***	Dabigatran	10–1,000 ng/mL (spiking, *n* = 10 donors)	None	None	Lindahl et al 18
			100–250 ng/mL (spiking, pooled plasma)	None	None	Van Blerk et al 20
		Rivaroxaban	120–290 ng/mL (spiking, pooled plasma)	None	Increase	Van Blerk et al 20
			11–1,090 ng/mL (spiking, pooled plasma)	None	Increase	Douxfils et al 36
			10–1,000 ng/mL (spiking, *n* = 10 donors)	None	Increase None if <50 ng/mL; no data between 50 and 100 ng/mL; significant if ≥100 ng/mL	Hillarp et al 24
			Not specified (ex vivo, *n* = 47 patients)	None	Increase	Mani et al 38
	***Hemosil IL***	Dabigatran	25–500 ng/mL (spiking, *n* = 10 donors)	None	None	Mannucci et al. 19
		Rivaroxaban	120–290 ng/mL (spiking, pooled plasma)	None	Increase	Van Blerk et al 20
		Apixaban	86.4–447.3 ng/mL (spiking, pooled plasma)	None	Increase Significant if ≥86.4 ng/mL	Douxfils et al 28
	***Hemosil Liquid AT***	Dabigatran	100–250 ng/mL (spiking, pooled plasma)	None	None	Van Blerk et al 20
			100–500 ng/mL (spiking, pooled plasma)	None	None	Vercruyssen et al 32
		Rivaroxaban	∼35–750 ng/mL (spiking, pooled plasma)	None	Increase Significant if ≥130 ng/mL	Gosselin et al 26
			100–500 ng/mL (spiking, pooled plasma)	None	Increase None if <100 ng/mL; no data between 100 and 250 ng/mL; significant if ≥250 ng/mL	Vercruyssen et al 32
		Apixaban	∼35–750 ng/mL (spiking, pooled plasma)	None	Increase ≥112 ng/mL	Gosselin et al 26
			41–225 ng/mL (spiking, pooled plasma)	None	Increase None if <41 ng/mL; no data between 41 and 94 ng/mL; significant if ≥94 ng/mL	Van Blerk et al 29
			100–250 ng/mL (spiking, pooled plasma)	None	Increase	Vercruyssen et al 32
		Edoxaban	∼25–470 ng/mL (spiking, pooled plasma)	None	Increase Significant if ≥269 ng/mL	Gosselin et al 26
			100–500 ng/mL (spiking, pooled plasma)	None	Increase	Vercruyssen et al 32
			100–500 ng/mL (spiking, pooled plasma)	None	Increase None if <100 ng/mL; no data between 100 and 250 ng/mL; significant if ≥250 ng/mL	Douxfils et al 35
	***IL Chromogenix***	Dabigatran	25–800 ng/mL (spiking, pooled plasma)	None	None	Bonar et al 21
		Rivaroxaban	7–638 ng/mL (spiking, pooled plasma)	None	Increase None if <87 ng/mL; no data between 87 and 211 ng/mL; significant if ≥211 ng/mL	Bonar et al 25
		Apixaban	8–501 ng/mL (spiking, pooled plasma)	None	Increase None if <118 ng/mL; no data between 118 and 296 ng/mL; significant if ≥296 ng/mL	Bonar et al 25
	***Innovance AT***	Dabigatran	100–250 ng/mL (spiking, pooled plasma)	None	None	Van Blerk et al 20
			25–800 ng/mL (spiking, pooled plasma)	None	None	Bonar et al 21
			71 [48–144] ng/mL (ex vivo, *n* = 27 patients)	DOAC-Stop ^TM^	None	Ząbczyk et al 89
		Rivaroxaban	120–290 ng/mL (spiking, pooled plasma)	None	Increase	Van Blerk et al 20
			∼35–750 ng/mL (spiking, pooled plasma)	None	Increase Significant if ≥100 ng/mL	Gosselin et al 26
			7–638 ng/mL (spiking, pooled plasma)	None	Increase None if <87 ng/mL; no data between 87 and 211 ng/mL; significant if ≥211 ng/mL	Bonar et al 25
			104 [45–334] ng/mL (ex vivo, *n* = 49 patients)	DOAC-Stop ^TM^	Increase	Ząbczyk et al 89
		Apixaban	8–501 ng/mL (spiking, pooled plasma)	None	Increase None if <118 ng/mL; no data between 118 and 296 ng/mL; significant if ≥296 ng/mL	Bonar et al 25
			∼35–750 ng/mL (spiking, pooled plasma)	None	Increase Significant if ≥112 ng/mL	Gosselin et al 26
			41–225 ng/mL (spiking, pooled plasma)	None	Increase None if <41 ng/mL; no data between 41 and 94 ng/mL; significant if ≥94 ng/mL	Van Blerk et al 29
			10–1000 ng/mL (spiking, *n* = 10 donors)	None	Increase	Hillarp et al 30
			93.5 [64–145] ng/mL (ex vivo, *n* = 54 patients)	DOAC-Stop ^TM^	Increase	Ząbczyk et al 89
		Edoxaban	∼25–470 ng/mL (spiking, pooled plasma)	None	Increase Significant if ≥38 ng/mL	Gosselin et al 26
			10–500 ng/mL (spiking, pooled plasma)	None	Increase	Hillarp et al 34
**FIIa-based assays**	***Berichrom ATIII***	Dabigatran	10–1,000 ng/mL (spiking, pooled plasma)	None	Increase	Lindahl et al 18
			25–500 ng/m (spiking, *n* = 10 donors)	None	Increase Significant if ≥125 ng/mL	Adcock et al 19
			100–250 ng/mL (spiking, pooled plasma)	None	Increase Significant if ≥100 ng/mL	Van Blerk et al 20
			25–800 ng/mL (spiking, pooled plasma)	None	Increase Significant if ≥100 ng/mL	Bonar et al 21
		Rivaroxaban	120–290 ng/mL (spiking, pooled plasma)	None	None	Van Blerk et al 20
			11–1,090 ng/mL (spiking, pooled plasma)	None	None	Douxfils et al 36
			10–1,000 ng/mL (spiking, *n* = 10 donors)	None	None	Hillarp et al 24
			7–638 ng/mL (spiking, pooled plasma)	None	None	Bonar et al 25
			82–532 ng/mL ( *n* = 4 commercial plasma)	Raw activated charcoal	None	Buckley et al 37
		Apixaban	8–501 ng/mL (spiking, pooled plasma)	None	None	Bonar et al 25
			179–647 ng/mL ( *n* = 4 commercial plasma)	Raw activated charcoal	None ( *n* = 4)	Buckley et al 37
		Edoxaban	10–500 ng/mL (spiking, *n* = 10 donors)	None	None	Hillarp et al 34
	***BiophenAT***	Dabigatran	80–500 ng/mL (spiking, pooled plasma)	None	Increase	Jacquemin et al 33
	***Cobas AT c***	Dabigatran	100–250 ng/mL (spiking, pooled plasma)	None	Increase Significant if ≥100 ng/mL	Van Blerk et al 20
	***Stachrom AT III***	Dabigatran	10–1,000 ng/mL (spiking, *n* = 10 donors)	None	Increase	Lindahl et al 18
			25–500 ng/mL (spiking, *n* = 10 donors)	None	Increase Significant if ≥125 ng/mL	Adcock et al 19
			100–250 ng/mL (spiking, pooled plasma)	None	Increase Significant if ≥100 ng/mL	Van Blerk et al 20
			25–800 ng/mL (spiking, pooled plasma)	None	Increase Significant if ≥100 ng/mL	Bonar et al 21
			4.7-943 ng/mL (spiking, pooled plasma)	None	Increase None if <50 ng/mL; no data between 50 and 100 ng/mL; significant if ≥100 ng/mL	Douxfils et al 22
			10–500 ng/mL (spiking, pooled plasma)	None	Increase None if <150 ng/mL; no data between 150 and 200 ng/mL; significant if ≥200 ng/mL	Kim et al 23
			71 [48–144] ng/mL (ex vivo, *n* = 27 patients)	DOAC Stop ^TM^	None	Ząbczyk et al 89
			Not specified (ex vivo, *n* = 30 patients)	DOAC Stop ^TM^	None	Favresse et al 68
		Rivaroxaban	120–290 ng/mL (spiking, pooled plasma)	None	None	Van Blerk et al 20
			10–1,000 ng/mL (spiking, *n* = 10 donors)	None	None	Hillarp et al 24
			7–638 ng/mL (spiking, pooled plasma)	None	None	Bonar et al 25
			40–430 ng/mL (spiking, pooled plasma)	None	None	Gerotziafas et al 27
			11–1,090 ng/mL (spiking, pooled plasma)	None	None	Douxfils et al 36
			Not specified (ex vivo, *n* = 47 patients)	None	None	Mani et al 38
			104 [45–334] ng/mL (ex vivo, *n* = 49 patients)	DOAC Stop ^TM^	None	Ząbczyk et al 89
			Not specified (ex vivo, *n* = 27)	DOAC Stop ^TM^	None	Favresse et al 68
			Not specified (ex vivo)	DOAC-Remove ^TM^	None	Cox-Morton et al 86
		Apixaban	8–501 ng/mL (spiking, pooled plasma)	None	None	Bonar et al 25
			86.4–447.3 ng/mL (spiking, pooled plasma)	None	None	Douxfils et al 28
			41–225 ng/mL (spiking, pooled plasma)	None	None	Van Blerk et al 29
			10–1,000 ng/mL (spiking, *n* = 10 donors)	None	None	Hillarp et al 30
			93.5 [64–145] ng/mL (ex vivo, *n* = 54 patients)	DOAC Stop ^TM^	None	Ząbczyk et al 89
			Not specified (ex vivo, *n* = 26 patients)	DOAC Stop ^TM^	None	Favresse et al 68
			Not specified (ex vivo)	DOAC-Remove ^TM^	None	Cox-Morton et al 86
		Edoxaban	100–500 ng/mL (spiking, pooled plasma)	None	None	Douxfils et al 35
			Not specified (ex vivo, *n* = 10 patients)	DOAC Stop ^TM^	None	Favresse et al 68
			Not specified (ex vivo)	DOAC-Remove ^TM^	None	Cox-Morton et al 86

Abbreviations: AT, antithrombin; DOAC, direct oral anticoagulant; IQR, interquartile range.

Notes: DOAC concentrations reported correspond either to the range (min–max) of DOAC concentration spiked or effectively measured in plasma samples prior to plasma treatment with DOAC removal agents or to the median [IQR]. When available, DOAC concentration to which no interference is observed (≤) or from which interference has been reported (≥) is specified. DOAC impact on AT measurement reported in the table results from the comparison between the measurement before and after DOAC addition in spiking experiment, or from the comparison between the measurement before and after plasma treatment with DOAC removal agent depending on the studies.

AT antigen measurement using immunological methods is not affected by DOACs, regardless the assay used or the DOAC compound. 7

##### Protein C and Protein S

PC and PS are vitamin K–dependent proteins that inactivate FVa and FVIIIa cofactors via the protein C pathway. Free PS acts as a cofactor of activated PC. Assessment of the PC anticoagulant activity or PS cofactor activity using clot-based assays allows the detection of all types of PC and PS deficiencies (i.e., either quantitative or qualitative). PC amidolytic activity can also be specifically measured by a chromogenic assay and free PS antigenic level can be measured by immunoassays, which are not affected by the presence of DOACs. 7 These methods cannot detect some rare qualitative deficiencies: type IIb PC deficiency and type II PS deficiency. However, because of the low specificity of clot-based assays linked to interferences, measurement of PC amidolytic activity and free PS antigenic levels is often performed as first-line assays to detect PC or PS deficiencies. 16 39 40 This should be clearly specified when they are used as first-line assays in thrombophilia testing.

Clot-based assays are based on activated partial thromboplastin time (aPTT) or Russell's viper venom (RVV), where both FX and FII are involved in clot formation. DOACs can thus prolong the clotting time, resulting in an overestimation of PC anticoagulant activity or PS cofactor activity levels, hence increasing the risk of missing a true deficiency. This was first described in DOAC-spiked samples. Similar to AT, these studies reported that DOAC interference on PC and PS activities is dose-dependent and varies as a function of the DOAC compound and the reagent used ( Tables 2 and 3 ). 19 21 25 26 30 PC activity measured with an aPTT-based assay is overestimated in plasma containing approximately 150 ng/mL or more dabigatran. 19 Despite the lower interference of oral FXa inhibitors with aPTT compared with dabigatran, some aPTT-based assays can be highly sensitive to these anticoagulant drugs. For instance, significant interference was observed with the Staclot® PC reagent (Stago) when apixaban plasma concentration was above 100 ng/mL, 30 whereas no effect was reported with Protein C reagent (Siemens). 30 With PS cofactor activity, all tested reagents appeared to be highly sensitive to the presence of dabigatran, rivaroxaban, and apixaban even at low plasma concentrations (around 35–50 ng/mL). 19 26 It should be noted that oral FXa inhibitors interfere more with PS cofactor activity than with PC anticoagulant activity. 26 30 Finally, Smock et al reported that the interference of rivaroxaban with PS cofactor activity and PC functional assays depended on the reagent used (RVV- *versus* aPTT-based method), RVV-based assays being more sensitive. 41

**Table 2:** DOAC interference with protein C anticoagulant activity testing

Methods	Assay used	DOAC	DOAC concentration Range	DOAC removal agent	Impact of DOAC on PC result Minimal C% without impact	Reference
***aPTT-based assays***	***Protein C Coag***	Dabigatran	25–500 ng/mL (spiking, *n* = 10 donors)	None	Increase None if <100 ng/mL; no data between 100 and 150 ng/mL; significant if ≥150 ng/mL	Adcock et al 19
		Rivaroxaban	∼35–750 ng/mL (spiking, pooled plasma)	None	Increase Significant if ≥222 ng/mL	Gosselin et al 26
		Apixaban	∼35–750 ng/mL (spiking, pooled plasma)	None	Increase Significant if >750 ng/mL	Gosselin et al 26
			10–1,000 ng/mL (spiking, *n* = 10 donors)	None	Increase	Hillarp et al 30
		Edoxaban	∼25–470 ng/mL (spiking, pooled plasma)	None	Increase Significant if ≥276 ng/mL	Gosselin et al 26
	***Staclot PC***	Rivaroxaban	Not specified (ex vivo)	DOAC-Remove ^TM^	Increase	Favre et al 81
		Apixaban	10–1,000 ng/mL (spiking, *n* = 10 donors)	None	Increase	Hillarp et al 30
			Not specified (ex vivo)	DOAC-Remove ^TM^	Increase	Favre et al 81

Abbreviations: aPTT, activated partial thromboplastin time; DOAC, direct oral anticoagulant; IQR, interquartile range; PC, protein C.

Notes: DOAC concentrations reported correspond either to the range (min–max) of DOAC concentration spiked or effectively measured in plasma samples prior to plasma treatment with DOAC removal agents or to the median [IQR]. When available, DOAC concentration to which no interference is observed (≤) or from which interference has been reported (≥) are specified. DOAC impact on PC measurement reported in the table results from the comparison between the measurement before and after DOAC addition in spiking experiment, or from the comparison between the measurement before and after plasma treatment with DOAC removal agent depending on the studies.

**Table 3:** DOAC interference with protein S cofactor activity testing

Methods	Assay used	DOAC	DOAC concentration Range	DOAC removal agent	Impact of DOAC on PS result Minimal C% without impact	Reference
***RVV-based assays***	***Cryocheck Clot S***	Dabigatran	25–500 ng/mL (spiking, *n* = 10 donors)	None	Increase (significant if ≥25 ng/mL)	Adcock et al 19
	***Protein S AC***	Apixaban	0–750 ng/mL (spiking, *n* = 10 donors)	None	Increase (significant if ≥50 ng/mL)	Hillarp et al 30
***aPTT-based assays***	***Staclot PS***	Dabigatran	25–500 ng/mL (spiking, *n* = 10 donors)	None	Increase (significant if ≥25 ng/mL)	Adcock et al 19
			10–500 ng/mL (spiking, pooled plasma)	None	Increase (significant if ≥50 ng/mL)	Kim et al 23
		Rivaroxaban	∼35–750 ng/mL (spiking, pooled plasma)	None	Increase (significant if ≥35 ng/mL)	Gosselin et al 26
			40–430 ng/mL (spiking, pooled plasma)	None	Increase	Gerotziafas et al 27
			11–1,090 ng/mL (spiking, pooled plasma)	None	Increase	Douxfils et al 36
			Not specified (ex vivo)	DOAC-Remove ^TM^	Increase	Favre et al 81
		Apixaban	∼35–750 ng/mL (spiking, pooled plasma)	None	Increase (significant if ≥471 ng/mL)	Gosselin et al 26
			86.4–447.3 ng/mL (spiking, pooled plasma)	None	Increase	Douxfils et al 28
			0–750 ng/mL (spiking, *n* = 10 donors)	None	Increase	Hillarp et al 30
			Not specified (ex vivo)	DOAC-Remove ^TM^	Increase	Favre et al 81
		Edoxaban	∼25–470 ng/mL (spiking, pooled plasma)	None	Increase (significant if ≥274 ng/mL)	Gosselin et al 26

Abbreviations: aPTT, activated partial thromboplastin time; DOAC, direct oral anticoagulant; IQR, interquartile range; PS, protein S; RVV, Russell's viper venom.

Notes: DOAC concentrations reported correspond either to the range (min–max) of DOAC concentration spiked or effectively measured in plasma samples prior to plasma treatment with DOAC removal agents or to the median [IQR]. When available, DOAC concentration to which no interference is observed (≤) or from which interference has been reported (≥) are specified. DOAC impact on PS measurement reported in the table results from the comparison between the measurement before and after DOAC addition in spiking experiment, or from the comparison between the measurement before and after plasma treatment with DOAC removal agent depending on the studies.

Conversely, DOACs do not interfere with amidolytic PC activity, and PC or PS antigene level measurements. 7

##### APC Resistance

APCR methods based on the aPTT are mostly affected by all DOACs. 18 23 24 26 28 While dabigatran highly increases APCR ratio with a risk of missing a real resistance, the slight increase induced by rivaroxaban and apixaban might not affect the final interpretation of the result (i.e., presence or absence of APCR). 42 43 Anti-Xa DOACs interference is lower (apixaban and edoxaban) or even absent (rivaroxaban) with the Pefakit APC-R factor V Leiden, which is a prothrombinase-based assay 18 24 34 36 44 45 46 ( Supplementary Table S1 , available in the online version only).

*On the basis of currently available evidence, we conclude that* :

1. *There is a risk of missing some AT, PC, or PS deficiencies and APCR when performing thrombophilia testing on plasma containing DOACs* .
2. *If AT, PC, PS, or APCR assays are prescribed, any current anticoagulant treatment or recent discontinuation should be mentioned. AT Cof activity can be measured using FIIa-based assays in patients receiving oral FXa inhibitors or FXa-based assays in patients receiving dabigatran. APCR can be replaced by genetic testing for FVL variant* .
3. *The lowest plasma concentration of DOACs that affects PC/PS clot-based activity or APCR is usually above the lower limit of quantification (LLOQ). It needs to be determined specifically for each DOAC and each instrument–reagent combination in clinical laboratories* .
4. *DOACs do not interfere with amidolytic PC activity and PS antigen level measurements. However, it should be clearly specified on the result reports that these methods do not allow the diagnosis of some rare qualitative deficiencies* .

#### Impact of DOACs on Lupus Anticoagulant Testing

Lupus anticoagulant is part of a heterogeneous group of aPL antibodies along with anti-β2GPI and aCL antibodies. They may be associated with arterial or VTE and/or pregnancy complications (mainly recurrent pregnancy loss, fetal death...), and define the antiphospholipid syndrome (APS) when persistent. 47 48 Paradoxically, LA inhibits phospholipid-dependent coagulation reactions in vitro *,* leading to prolongation of clotting times. 49 The International Society on Thrombosis and Haemostasis recommends the use of two tests based on different analytical principles for a reliable LA diagnosis. 50 51 LA diagnosis is considered positive when at least one of those tests is positive. The most widely used assays in clinical laboratories associate the dilute RVV time (dRVVT) with a LA-sensitive aPTT. Assays are performed in the presence of phospholipids added at low (screen assay) or high (confirm assay) concentration. Patient screen and confirm results are expressed as ratios to reference plasma results. Final results are usually expressed as screen ratio/confirm ratio, called a normalized ratio. In addition, a mixing test (i.e., a 1:1 mix of patient and pooled normal plasma) is recommended to improve the discrimination between the presence of an inhibitor and coagulation factor deficiencies such as those occurring in VKA patients. Testing should be repeated at least 12 weeks after an initial LA positive result to establish the diagnosis of APS. All DOACs have the potential to interfere with LA assays, but the degree of interference varies depending on the drug, its concentration, reagent, and diagnostic strategy. Investigation with solid phase assays for aPL, such as aCL and anti-β2GPI, is not addressed in this review as DOACs do not interfere with these assays.

##### dRVVT-based Assays

Dabigatran significantly prolongs dRVVT screen and confirm clotting times, but the interference with the normalized dRVVT ratio remains uncertain. Some studies concluded false-positive results, 21 52 53 54 55 56 with a lowest interfering concentration being less than 20 ng/mL, while others described a parallel prolongation of dRVVT screen and confirm clotting times, therefore without significant impact on the final normalized dRVVT ratio. 57 58 59

Since the first report of a high prevalence of abnormal normalized dRVVT ratio in patients receiving rivaroxaban, 60 many studies using spiked samples or ex vivo patient samples have confirmed this finding. The prolongation of dRVVT screen and confirm clotting times measured in spiked samples was concentration dependent and seemed to be significant even at rivaroxaban concentrations below the LLOQ of the various commercially available specific anti-Xa assays used in hemostasis laboratories. 54 55 58 Because dRVVT confirm clotting times are often shorter than dRVVT screen clotting times, there is a significant risk of falsely increased normalized dRVVT ratio, resulting in false-positive results. 25 53 54 55 56 58 61 62 63 64 The minimal concentration of rivaroxaban below which there would be no significant interference is difficult to determine, first because most of the studies were not designed for that purpose, and second because the sensitivity of the dRVVT reagents to rivaroxaban varies significantly. 59 The rate of false-positive results with the dRVVT normalized ratio at rivaroxaban concentration below 50 ng/mL varied significantly between studies. Indeed, it was estimated to be between 30 and 90% of the samples in some studies, 54 58 63 while in others it was very low or even null. 25 52 59 65

Fewer data are available for edoxaban. Low concentrations of edoxaban (i.e., 61 ng/mL corresponding to trough concentrations observed with standard dosing regimens) increased the risk of false-positive dRVVT results in spiked samples. Similar to rivaroxaban, edoxaban significantly prolonged dRVVT screen, and to a lesser extent the dRVVT confirm clotting times, leading to normalized ratios above the cutoff. 66 67

The prolongation of dRVVT screen clotting times with apixaban is less pronounced than with the other three DOACs. Indeed, apixaban concentrations leading to false-positive dRVVT screen results were mostly described above 30 ng/mL, 25 52 56 58 59 except in one study. 54 The rate of false-positive dRVVT normalized ratio was usually low, between 7 and 20%, 54 55 58 64 if any, 25 52 56 59 but was found to be 40% in one study. 63 Several studies showed that apixaban has a more pronounced effect on dRVVT confirm than on dRVVT screen, leading to a risk of false-negative dRVVT normalized ratio result. 52 56 58 59 Specifically designed studies are required to refute or confirm these findings.

##### aPTT-based Assays

Results obtained with aPTT-based assays are more heterogeneous than those obtained with the dRVVT-based reagents.

Dabigatran significantly prolonged several aPTT-based screen clotting times, PTT LA (Stago), SynthASil (Werfen), and SCT screen (Werfen), 53 54 55 58 yet to a lesser extent than with dRVVT-based assays. Confirm clotting times with Actin-FS (Siemens), APTT-SP, or SCT (Werfen) were less prolonged than the screen clotting times, increasing the risk of false-positive normalized ratio results. 53 55 58

In rivaroxaban samples, van Os et al showed comparable prolongation of aPTT screen (PTT LA, Stago) and confirm (Actin FS, Siemens) clotting times, resulting in a normalized ratio that was quite unchanged. 61 Other studies reported no or little effect on PTT LA results. 54 55 No false-positive results were reported with Staclot LA (Stago) 60 and no difference was observed between samples collected at trough or peak concentrations after rivaroxaban intake when LA testing was performed using the Mixcon LA assay (Instrumentation Laboratory). 66 Two studies reported prolonged SCT screen clotting times (Werfen) (i.e., positive screen results) in the presence of rivaroxaban 62 63 ; one reported normal normalized ratio (i.e., negative LA results), 62 and the other reported a 40% increase of positive normalized ratios (i.e., positive LA results). 63 No false-positive results were observed with the Actin FSL/Actin FS ratio (Siemens) in one study, 64 while 17% of false-positive results were observed in another one. 63 The rate of false-positive results with aPTT SynthASil (Werfen) and aPTT-SP (Werfen) increased in a concentration-dependent manner in rivaroxaban samples. 58

Spiking experiments revealed falsely prolonged aPTT-based screen clotting times at edoxaban plasma concentrations equal or slightly higher than those interfering with dRVVT assays. On the flip side, confirm clotting times were unaffected leading to false-positive normalized ratios. 67 68

Studies evaluating the interference of apixaban with aPTT-based assays yielded controversial results. While no prolonged PTT LA clotting time was observed at plasma concentration up to 178 ng/mL in one study 55 and up to 600 ng/mL in another, 56 apixaban (up to 150 ng/mL) prolonged PTT LA clotting times in a concentration-dependent manner in a third, yet the ratios remained below the local cutoff. 54 Using SynthASil, approximately 10% of apixaban patient samples (up to 120 ng/mL) were false positive. 58 With the SCT Screen/Confirm and Actin FSL/FS combinations, the rate of false-positive results of the normalized ratio was estimated to be 30 and 20% of the apixaban patient samples, respectively. 63 While no impact of apixaban (up to 370 ng/mL) was observed on Cephen LS screen or Cephen LS/Cephen normalized ratios in patient samples, Cephen confirm ratios seemed to be higher than the corresponding Cephen LS screen ratios which could result in false-negative results. 69 This remains to be appropriately investigated in future studies.

*On the basis of currently available evidence, we conclude that* :

1. *There is a high risk of false-positive LA results in plasma containing DOACs, particularly rivaroxaban regardless of the assay used for LA testing* .
2. *If LA testing is prescribed, any current or recently discontinued anticoagulant treatment must be mentioned. The lowest DOACs plasma concentration that interferes with dRVVT tests remains unknown. Commercial anti-Xa and anti-IIa assays available in hemostasis laboratories may not be sensitive enough to detect such low interfering concentrations* .
3. *Available data on aPTT-based assays in the presence of DOACs are too heterogeneous to allow any conclusion regarding the plasma concentration threshold below which no interference with LA results is observed* .

### DOAC Removal Agents

#### DOAC Removal Agent Types

The use of a device that can remove DOAC compounds from blood/plasma samples in vitro should be considered to minimize the risk of false-negative or false-positive results. Specific antidotes such as idarucizumab for dabigatran and andexanet alfa for rivaroxaban and apixaban have been tested 33 70 71 but they are expensive and not widely available, and therefore cannot be used in routine clinical laboratories for diagnostic purposes.

Other products have been specifically developed for laboratory use. 72 Four of them are based on activated charcoal: DOAC-Stop™ (Haematex) and DOAC-Remove™ (5-Diagnostic AG), both available as tablets, Carbomix ^®^ (Norit Nederland B.V.) as powder, and DOAC-Stop Liquid™ (Haematex), a liquid form of DOAC-Stop™. 73 Activated charcoal consists of porous elemental carbon with a large internal surface area. A fourth DOAC removal device consisted in a single and ready-to-use filtration cartridge called DOAC-Filter® (Diagnostica Stago). The hydrophilic–hydrophobic balance of the solid phase was determined to specifically trap DOACs. Although interesting results were published with DOAC Filter®, 74 75 76 77 its commercialization was cancelled in 2022 due to the limited volume of filtered plasma, hence requiring two filters for a full panel of LA testing, and the relatively high manufacturing cost. Hence, in this review, we will focus on the potential usefulness and limits of the currently available in vitro DOAC removal agents, all charcoal-based, namely, DOAC-Stop ^TM^ (DS), DOAC-Stop Liquid ^TM^ (DS-L), and DOAC-Remove ^TM^ (DR), for reliable thrombophilia testing in DOAC plasma samples.

#### Removal Efficiency of Activated Charcoal on DOACs in Plasma

Although the gold standard method for measuring DOAC plasma concentration is the high-performance liquid chromatography coupled with electrospray ionization tandem mass spectrometry (HPLC-MS/MS), this method is not used in daily clinical practice nor widely available. Instead, clotting or chromogenic assays using specific standard calibrators and controls are generally used to quantify DOAC plasma concentrations 78 and were generally employed to assess the removal efficiency of activated charcoal on DOACs in plasma ( Table 4 ).

**Table 4:** DOAC removal efficacy of activated charcoal in treated patient samples

Activated charcoal agent	DOAC type	Assays for DOAC plasma concentration measurement	DOAC concentration before activated charcoal Median (range)	LLOQ	Number of incomplete neutralization/(%)	Reference
DOAC-Remove ^TM^	Dabigatran	dTT (Hyphen)	135 (<20–792) ng/mL	<20 ng/mL	0% (0/24)	Jourdi et al 79
		dTT (Hyphen)	199–538 ng/mL	<30 ng/mL	0% (0/2)	Favre et al 81
	Rivaroxaban	Specific Liquid anti-Xa (Stago)	107 (<20–501) ng/mL	<20 ng/mL	**2% (1/48)**	Jourdi et al 79
		Specific Liquid anti-Xa (Stago)	121 (34–359) ng/mL	<25 ng/mL	0% (0/41)	Melicine et al 80
		Specific Liquid anti-Xa (Stago)	140 (57–922) ng/mL	<25 ng/mL	**4% (1/27)**	Favre et al 81
		Specific Liquid anti-Xa (Stago)	Not precised	<15 ng/mL	0% (0/11)	Raulet-Bussian et al 82
		LMWH Liquid anti-Xa (Stago)	1.65 (0.91) IU/mL	<0.1 IU/mL	0% (0/29)	Cox-Morton et al 86
	Apixaban	Specific Liquid anti-Xa (Stago)	94 (<20–479) ng/mL	<20 ng/mL	**18% (9/49)**	Jourdi et al 79
		Specific Liquid anti-Xa (Stago)	116 (31–396) ng/mL	<23 ng/mL	0% (0/42)	Melicine et al 80
		Specific Liquid anti-Xa (Stago)	165 (80–634) ng/mL	<23 ng/mL	**6% (1/18)**	Favre et al 81
		Specific Liquid anti-Xa (Stago)	Not specified	<15 ng/mL	0% (0/9)	Raulet-Bussian et al 82
		LMWH Liquid anti-Xa (Stago)	1.67 (0.32) IU/mL	< 0.1 IU/mL	0% (0/3)	Cox-Morton et al 86
	Edoxaban	LMWH Liquid anti-Xa (Stago)	0.32 (0.02) IU/mL	< 0.1 IU/mL	0% (0/2)	Cox-Morton et al 86
DOAC-Stop ^TM^	Dabigatran	Ecarin chromogenic assay (Stago)	2–406 ng/mL	<27 ng/mL	0% (0/40)	Favresse et al 68
		TT (Instrumentation laboratory)	70–300 seconds *visually deduced*	<20 seconds	0% (0/3)	Baker et al 87
		dTT (Werfen)	161 (74–231) a ng/mL	<10 ng/mL	0% (0/39)	Tripodi et al 85
		dTT (Werfen)	108.9 (<20–315) ng/mL	<20 ng/mL	0% (0/23)	Novelli et al 84
		dTT (Werfen)	82 (47–128) ng/mL	Not specified	0% (0/4)	Savola et al 77
		dTT (Hyphen)	30–250 ng/mL	<10 ng/mL	0% (0/9)	De Kesel et al 67
		dTT (Hyphen)	23–373 ng/mL	<21 ng/mL	0% (0/6)	Linskens et al 76
	Rivaroxaban	Specific Liquid anti-Xa (Stago)	35–604 ng/mL	<21 ng/mL	**7% (1/14)**	De Kesel et al 67
		Specific Liquid anti-Xa (Stago)	7–456 ng/mL	<25 ng/mL	0% (0/42)	Favresse et al 68
		Specific Liquid anti-Xa (Stago)	27–508 ng/mL	< 21 ng/mL	0% (0/20)	Linskens et al 76
		Specific Liquid anti-Xa (Werfen)	78 (16–192) a ng/mL	<10 ng/mL	0% (0/55)	Tripodi et al 85
		Specific Liquid anti-Xa (Werfen)	75.4 (<20–320) ng/mL	<20 ng/mL	0% (0/30)	Novelli et al 84
		Specific Liquid anti-Xa (Werfen)	106 (82–265) ng/mL	Not specified	0% (0/7)	Savola et al 77
		Biophen Heparin LRT (Hyphen)	165 (100–231) b ng/mL	<17 ng/mL	**5% (1/20)**	Platton et al 83
		LMWH Liquid anti-Xa (Instrumentation laboratory)	0.1–3.8 IU/mL *visually deduced*	<0.1 IU/mL	0% (0/26)	Baker et al 87
	Apixaban	Specific Liquid anti-Xa (Stago)	43–688 ng/mL	<20 ng/mL	0% (0/9)	De Kesel et al 67
		Specific Liquid anti-Xa (Stago)	10–316 ng/mL	<15 ng/mL	0% (0/38)	Favresse et al 68
		Specific Liquid anti-Xa (Stago)	51–279 ng/mL	<20 ng/mL	0% (0/4)	Linskens et al 76
		Specific Liquid anti-Xa (Werfen)	187 (94–282) a ng/mL	<10 ng/mL	**13% (6/47)**	Tripodi et al 85
		Specific Liquid anti-Xa (Werfen)	172.4 (32–591) ng/mL	<15 ng/mL	0% (0/31)	Novelli et al 84
		Specific Liquid anti-Xa (Werfen)	162 (73–572) ng/mL	Not specified	>25 ng/mL: 14% (1/7)	Savola et al 77
		LMWH Liquid anti-Xa (Instrumentation laboratory)	0.2–4.0 IU/mL *visually deduced*	<0.1 IU/mL	0% (0/38)	Baker et al 87
		Biophen Heparin LRT (Hyphen)	154 (95–214) b ng/mL	<13 ng/mL	**10% (2/20)**	Platton et al 83
	Edoxaban	Specific Liquid anti-Xa (Stago)	23–344 ng/mL	<20 ng/mL	0% (0/11)	De Kesel et al 67
		Specific Liquid anti-Xa (Stago)	21–354 ng/mL	<20 ng/mL	0% (0/15)	Favresse et al 68
		Specific Liquid anti-Xa (Stago)	22–239 ng/mL	<20 ng/mL	0% (0/5)	Linskens et al 76
		Specific Liquid anti-Xa (Werfen)	46 (30–249) ng/mL	<40 ng/mL <10 ng/mL	0% (0/47) **51% (24/47)**	Tripodi et al 85
		Specific Liquid anti-Xa (Werfen)	152 (35–283) ng/mL	Not specified	0% (0/4)	Savola et al 77
		Specific Liquid anti anti-Xa (Werfen)	105 (<15–304) ng/mL	<15 ng/mL	0% (0/21)	Novelli et al 84

Abbreviations: CI, confidence interval; DOAC, direct oral anticoagulant; dTT, dilute thrombin time; IQR, interquartile range; LMWH, low-molecular-weight heparin.

a median (IQR)

b mean (95% (CI))

After treatment of plasma samples with DR, DOAC concentrations were below the LLOQ in at least 100% dabigatran, 79 96% rivaroxaban, and 82% apixaban patient samples 37 79 80 81 82 ( Table 4 ). In a fourth study performed in spiked normal pooled plasma (Pool Norm, Stago, REF 00539), the percentage of samples with DOAC concentrations below the LLOQ post treatment with DR was not determined. However, DR reduced DOACs concentration (up to 600 ng/mL) to below 30 ng/mL for apixaban and rivaroxaban but to higher concentrations (i.e., 83 ± 29 ng/mL) for dabigatran. 56

In a study with DS, residual concentrations were below the LLOQ in all samples except in 1 out of 20 rivaroxaban (95%) and 2 out of 20 apixaban patient samples (90%) 83 ( Table 1 ). In a second study, residual DOAC concentrations were below the LLOQ for all samples except 3 out of 14 dabigatran (21.4%) and 1 out of 11 rivaroxaban (9%) patient samples. 67 In three other studies, all samples were below the LLOQ, yet different LLOQ values were reported. 68 76 84 In a sixth study, DS reduced DOAC concentrations below 40 ng/mL in 100% of samples, and below 10 ng/mL in 100% dabigatran, 100% rivaroxaban, 87% apixaban, and 49% edoxaban samples. 85 Another study reported 100% neutralization of DOAC for dabigatran, rivaroxaban, and edoxaban but one out of seven apixaban (14%) remained >25 ng/mL after treatment with DS. 77 DS reduced all three DOACs concentration (up to 600 ng/mL) to a mean level below 30 ng/mL in an eighth recent study without determining if incomplete neutralization was observed in some samples. 56

In two additional studies (one with DR and one with DS), residual DOAC was detected using low-molecular-weight heparin (LMWH) anti-Xa activity (for oral FXa inhibitors) or thrombin time (TT, for dabigatran). An anti-Xa activity below 0.1 IU/mL and a normal TT were observed in 100% of the treated samples. 86 87

Overall, residual DOAC plasma concentrations following sample treatment with activated charcoal were often below the LLOQ of specific anti-IIa and anti-Xa assays, which varies from one study to another. Activated charcoal agents are often more effective to reduce dabigatran and rivaroxaban than apixaban concentrations ( Table 5 ). It should be noted that residual DOAC concentration (i.e., post-DOAC removal agents) is not always positively correlated with baseline concentration and that a double dose of pellets did not always allow complete DOAC neutralization. 67

**Table 5:** Pros and cons of available diagnostic strategies for thrombophilia testing in DOAC-treated patients

	Advantages	Limits/Drawbacks
**DOAC discontinuation**	• No additional cost	• Possible thrombotic risk • Possible hemorragic risk in case of LMWH bridging therapy • Possible risk of interference with residual DOAC concentration (particularly with LA testing)
**DOAC-insensitive assays/alternative assays**	• No change of treatment	• Not widely available (for LA testing) • Not standardized (for LA testing) • Can miss some qualitative deficiencies (for PC/PS)
**DOAC removal agents**	• No change of treatment	• Local validation required • Increase in cost (costs of the removal agent and DOAC concentration measurement) • Time-consuming • Do not warrant the absence of interference (particularly for LA testing)

Abbreviations: DOAC, direct oral anticoagulant; LA, lupus anticoagulant; LMWH, low-molecular-weight heparin; PC, protein C; PS, protein S.

*On the basis of currently available evidence, we conclude that* :

1. *Activated charcoal could be useful to remove DOACs from plasma prior to thrombophilia testing, but may not be completely effective, particularly with apixaban* .
2. *Due to the risk of incomplete DOAC adsorption, residual DOAC concentration should be determined after in vitro treatment of sample with activated charcoal, if there is any concern about efficacy* .

#### Effect of Activated Charcoal Agents on Hemostasis Testing in the Absence of DOAC

Several studies reported an impact of the pretreatment of plasma with charcoal products on coagulation assays. DR was shown to significantly shorten aPTT and TT ( *p* < 0.001) in 20 non-anticoagulated LA negative patient samples. This contrasted with a slight but significant increase in aPTT results and a small decrease in fibrinogen levels following sample treatment with one or two DR pellets. 88 Effect on fibrinogen level was confirmed in the study by Skaugen et al, along with a consistent weak prolongation (<0.5 seconds) of the prothrombin time (PT) in five normal donor samples after DR treatment 93 In parallel, no significant variations were observed in PT, aPTT, as well as FVII, FVIII, and FX clotting activities with DS. 67 73 83 84 88 In contrast, Riva et al reported significant reduction of FVIII, FIX, FX, FXI, and FXII levels without any relevant increase in the aPTT. 94 This might be explained by the different sensitivity of the reagents used. Both DOAC removal agents enhanced thrombin generation and lowered TFPI concentration in a dose-dependent manner (one vs. two tablets) in pooled normal plasma samples. 88 The neutrality of DOAC removal agents should be verified before its implementation in clinical practice.

##### Inherited Thrombophilia Testing

Four studies have evaluated the effect of activated charcoal agents on natural coagulation inhibitor assays in DOAC-free plasma samples. No impact on AT Cof was observed with either DS 68 88 or DR. 88 Concerning PC and PS clot-based activities, only the impact of DR was tested in DOAC-free plasma samples. 81 82 No significant effect on inhibitor level was observed. Two other studies showed no impact of DS and DR on PC amidolytic activity but inconsistent results for free PS antigen level in DOAC-free control plasma, despite the same method was used in both studies. 68 88

Two studies reported no potential impact on APCR testing when using DOAC-Stop. 57 68

##### LA Testing

As with inherited thrombophilia, it was essential to determine whether removal agents interfered with LA assays in the absence of DOAC. Three studies evaluated the impact of DR on LA assays. It had no significant effect on dRVVT and LA sensitive aPTT assays (Staclot, Stago) in pooled normal plasma (Pool Norm, Stago, REF 00539), 56 nor on dRVVT and LA sensitive aPTT assays (APTT-SP, Werfen) in 8 LA-positive and 10 LA-negative patient samples. 86 This was confirmed in 10 LA-negative, 10 low LA-positive (i.e., normalized dRVVT ratio between 1.20 and 1.50), and 10 high LA-positive (i.e., normalized dRVVT ratio higher than 1.50) samples in our French multicenter study. 79

For DS, six studies evaluated its potential interference with LA testing in the absence of DOAC therapy. No significant changes were observed in dRVVT, 56 68 73 83 84 87 SCT screen and confirm 84 as well as Staclot LA results in LA-negative or -positive patients (65.73–75.78) or in pooled normal plasma (Pool Norm, Stago, REF 00539). 56 Conversely, dRVVT conclusions changed from positive to negative in eight out of 63 samples (12.7%), whereas two negative results became positive (3.2%). 67 No significant effect was observed on Staclot LA 67 or PTT LA assays. 67 73 Also, one in five weakly positive LA samples became negative after treatment with DS in another study. 83 Altered dRVVT conclusions resulted from small changes in clotting times near the cutoff. Thus, these differences in clotting times were within the assay's analytical variability. 67 This might also be explained by the fact that DS could absorb endogenous proteins and thus affect clotting assays.

*On the basis of currently available evidence* :

1. *We conclude that DOAC removal agents may not be completely neutral with respect to LA testing in the absence of DOAC, particularly in weakly positive LA samples (i.e., close to the cutoff)* .
2. *We advise against treating DOAC-free samples with activated charcoal* .
3. *We advise against treating normal pool plasma sample used to calculate the screen, confirm, and normalized ratios with activated charcoal* .

### Usefulness of Activated Charcoal for Inherited Thrombophilia Testing in Samples from DOAC-treated Patients

Several studies have evaluated the effect of DOAC removal agents based on activated charcoal on inherited thrombophilia testing in patients treated with DOACs. DS 68 89 and DR 86 did not impact FXa- (in dabigatran samples) or FIIa-based (in oral FXa inhibitors samples) AT Cof activity, PC amidolytic activity, or free PS antigen levels after in vitro DOAC neutralization ( Tables 1 – 3 ). However, as expected, AT Cof based on the anti-Xa assay was overestimated in oral FXa inhibitor containing samples, with a median decrease in AT Cof activity of 18% observed after in vitro plasma treatment with DS (median AT Cof activity at baseline, 83.5%; interquartile range [IQR] 66–143% *versus* 65.5%; IQR 57–75% after DS, *p* = 0.005) ( Table 1 ). 89 However, neither Favresse et al nor Ząbczyk et al showed a significant change in AT Cof activity following in vitro treatment with DS of dabigatran patient plasma samples despite the use of a FIIa-based AT assay (STA-Stachrom AT III, Stago or Siemens, respectively). 68 89 This could be explained by low dabigatran plasma concentration in these studies (with median [Min; Max] at 73.5 ng/mL [2; 406] 68 and median [IQR] at 71 ng/mL [48; 144], 89 respectively). Indeed, in spiked samples, dabigatran interfered with AT Cof activity at concentrations higher than 125 and 100 ng/mL, respectively. 19 21 The study by Ząbczyk et al was the first to include samples from true AT-deficient patients. Out of the 10 AT-deficient patients 3 already presented decreased AT Cof activity results while receiving oral FXa inhibitor; in the others, the deficiency was observed only after in vitro sample treatment with DS. 89 No such data on DR or DS-L have been reported in the literature.

No alternative assay can replace clot-based activity measurement used to screen for all types of PC or PS deficiencies. DOACs interfere with these assays at concentrations higher than 30 ng/mL. Therefore, if in vitro sample treatment with activated charcoal agents decreases DOAC plasma concentration below the aforementioned threshold, it allows reliable use of these clot-based assays. Indeed, DR has proven its efficacy to mitigate oral FXa inhibitors interference with aPTT-based PC assay in patient samples ( Table 2 ). 81 82 Similarly, DR has also proven its efficacy to minimize oral FXa inhibitor overestimating interference with PS anticoagulant activity measured using aPTT-based assay in patient samples ( Table 3 ). 81 Notably, a positive correlation was observed between the degree of PS activity decrease after in vitro sample treatment with DR and the initial oral FXa inhibitor concentration. 81 Interestingly, PS activity measurement after DR revealed a true PS qualitative deficiency that would have been missed if only free PS antigen had been measured (92% before versus 53% after charcoal) in a patient with a genetically confirmed qualitative PS deficiency. 81 There is no study evaluating the utility of DS or DS-L in mitigating DOAC interference with PC or PS clot-based assays.

Three studies have evaluated the effect of DS 57 68 and DR 90 on APCR testing in patients treated with DOACs. Both the agents circumvent DOAC interference with APCR assays in DOAC-treated patients.

*On the basis of currently available evidence* :

1. *For AT cofactor activity measurement in DOAC-treated patients, we recommend choosing the appropriate assay (FXa- or FIIa-based assay) over in vitro DOAC neutralization with activated charcoal* .
2. *For PC and PS clot-based anticoagulant activities, in vitro DOAC neutralization with activated charcoal may be necessary to obtain reliable results* .
3. *The neutrality of DOAC removal agents with respect to PC and PS clot-based assays and APCR (whenever performed) should be verified locally in each laboratory before its implementation in clinical practice* .
4. *If DOAC concentration is below the interference cutoff (for locally determined, see Impact of DOACs on inherited thrombophilia screening), in vitro sample treatment with activated charcoal should not be performed* .
5. *If DOAC concentration is above the interference cutoff (for locally determined, see Impact of DOACs on inherited thrombophilia screening), in vitro sample treatment with activated charcoal should be performed, and residual DOAC plasma concentration should be determined before measuring PC and/or PS anticoagulant activities* .
6. *If the interference cutoff is unknown or below the LLOQ of the specific anti-Xa or anti-IIa assay, results should be interpreted with caution* .

### Usefulness of DOAC Removal Agents for LA Testing in Samples from DOAC-treated Patients

Nine publications evaluating the utility of DS for reliable LA diagnosis in patients taking DOACs were included in this review, 52 67 68 77 83 84 87 91 92 four with DR 79 81 86 94 and one with activated charcoal powder (Carbomix ^®^ 20 mg/mL). 95

#### Dabigatran

Dabigatran prolonged aPTT, SCT, and dRVVT. False-positive dRVVT screen was observed at dabigatran concentration above 23 ng/mL in patient samples. 68 False-positive PTT-LA was observed starting at 33 ng/mL in patient samples. 68 Potential dabigatran interference with LA results persisted in very few patient samples after treatment with activated charcoal-based agents: in 2/39 patients with DS (LA testing using dRVVT) 85 and in 1/21 (dRVVT), 79 and 5/23 (dRVVT and SCT) 84 with DR.

#### Rivaroxaban

False-positive dRVVT results were observed at rivaroxaban concentrations above 35 ng/mL. 68 False-positive PTT LA results were also observed at 23 ng/mL in patient samples. 68 After in vitro plasma treatment with DR, positive LA conclusions persisted in 3/29 (dRVVT) 86 and 7/43 (dRVVT) 79 patient samples. After DS, positive LA conclusions were observed in 3/55 (dRVVT) and 2/55 (SCT) samples, 85 2/20 (dRVVT) samples, 83 and 3/26 samples (including 2 likely true LA) (dRVVT and SCT). 87 In two other studies, all patient samples were LA negative after activated charcoal procedure. 68 81

Three studies have assessed activated charcoal utility in true LA-positive patients receiving DOACs: four LA-positive patients taking rivaroxaban still had LA-positive results, three after sample treatment with DR, 79 and one after DS. 67 Eight LA-positive patients taking an oral FXa inhibitor remained positive after DR. 86

#### Edoxaban

Compared with other DOACs, fewer data are available with regard to the usefulness of activated charcoal-based removal agents for reliable LA testing in edoxaban patient samples. Five out of seven initially positive LA results turned out ot be negative after in vitro plasma treatment with DS, 67 with one of the two remaining LA positive samples belonging to a patient having a high clinical probability of antiphospholipid syndrome. 67 In a second study, one patient on edoxaban was initially classified as LA negative (using SCT assay) and turned slightly positive following in vitro sample treatment with DS. 85 Inversely, 1 out of 14 patient samples in one study and 3 out of 4 in another, with initially LA-positive results, turned out to be LA negative after in vitro sample treatment with DR 96 and DS, 77 respectively. In a fifth study, LA conclusion (based on dRVVT and SCT assays) changed from negative to positive in 1 out of 21 patients receiving edoxaban. 84

#### Apixaban

Apixaban has the least effect on dRVVT and aPTT-based assays compared with rivaroxaban, edoxaban, and dabigatran. However, residual concentration above the LLOQ is most often observed with this DOAC after charcoal procedures, potentially leading to false-positive results. 79 83 85 Positive dRVVT screening has been observed in patient samples with plasma concentration above 20 ng/mL. 68 Positive PTT-LA has also been observed above 26 ng/mL. 68 After DR treatment, a positive LA conclusion was observed in up to 24% of samples tested (dRVVT). 79 In another study, 100% of the results became negative. 86 After DS treatment, positive LA conclusions were observed in 1/47, 85 1/38, 87 and 6/31 84 (dRVVT and SCT) of patient samples, respectively. In contrast, no false-positive result was observed in another study. 68 False-negative dRVVT could also be observed, particularly in patients treated with apixaban, due to the greater interference of this DOAC at residual concentration (i.e., after sample treatment with activated charcoal) with the confirm assay compared with the screen assay. 97

*On the basis of currently available evidence* :

1. *We conclude that negative LA screen results are reliable in samples from patients receiving DOACs not treated with activated charcoal. In such cases, no LA confirm assays should be performed* .
2. *We conclude that negative LA screen results are reliable in samples from patients receiving DOACs treated with activated charcoal. In such cases, LA confirm assays should not be performed* .
3. *We advise against performing confirm assays in case of positive screen results in samples treated with activated charcoal due to the difficulty to distinguish between true and false-positive results* .
4. *We advise, in case of persistent positive screen results after in vitro sample treatment with a removal agent, repeating LA testing after DOAC discontinuation for at least 72 h (to be adapted on a case-by-case basis, depending on the thrombotic risk of the patient)* .

## Discussion

DOACs affect differently acquired and inherited thrombophilia testing. Although false-positive results are the main risk with LA detection in samples containing DOACs, certain assays may overestimate inhibitory activity in the presence of DOACs, leading to a potential failure to detect true deficiencies in AT, PC, and/or PS. Because of the high sensitivity of LA detection assays to DOACs, it cannot be ruled out that residual DOAC concentrations (even below the LLOQ of specific anti-Xa and anti-IIa assays) may still impact LA testing, potentially leading to a false-positive LA result despite the use of DOAC removal agents. The difficulty lies in being able to confirm/infirm the presence of true LA. For all these reasons, reporting ongoing DOAC therapy, and even any other anticoagulant, is mandatory when prescribing thrombophilia testing.

Many strategies were previously proposed to circumvent DOAC interference with thrombophilia testing. They are summarized in Table 5 . Choosing appropriate assays might circumvent DOAC interference with thrombophilia testing such as reagent based on FIIa or FXa assays for AT, provided these tests are both available. For PC and PS, quantitative deficiencies, either inherited or acquired, and some qualitative inherited deficiencies can be detected by chromogenic PC activity or free PS antigenic level measurements, which are not affected by DOACs, 40 whereas the detection of rare qualitative deficiencies of PC and PS relies on the use of clot-based assays. Genotyping cannot be proposed as a first-line alternative for patients receiving DOACs due to its cost. For LA detection, tests that have a lower sensitivity toward DOACs such as Taipan Snake Venom Time/Ecarin time have been developed, 14 but they are neither widely available nor standardized. Although mixing test might circumvent VKA interference with LA measurement, this does not apply for DOAC. The most useful alternative is thus the in vitro use of DOAC removal agents, hence the aim of our review. Available data support the use of these systems to overcome DOAC interference in thrombophilia testing, but several issues remain unresolved. Both the DOAC plasma concentration interfering cutoff and the impact of DOAC removal agents on thrombophilia testing in the absence of DOAC need to be assessed locally in each laboratory.

A critical review of the literature unveils significant heterogeneity in published data, particularly with respect to analytical features, interpretation strategies, and threshold used. Moreover, although various activated charcoal agents were used according to manufacturer's instructions, many analytical differences were observed among the published studies. These differences were mainly related to the plasma volume (0.5 to 2.0 mL per pellet), the treatment of fresh or frozen plasma, incubation time (5 to 10 minutes), centrifugation time (1 to 10 minutes), and centrifugation speed (2,000 to 7,000 g). Thus, it remains to be determined whether such differences may explain the discrepancies between the published studies. Moreover, LA result interpretation also differed among studies: dRVVT (cutoff for normalized ratio 1.20 or 1.10, with or without mixing study), aPTT (with or without confirmatory test), SCT (screen with or without confirmation), etc. In the case of persistently positive results following in vitro treatment of specimen with a DOAC removal agent, important information is often missing that could compromise the confirmation of the LA diagnosis (e.g., solid phase assays for aPLs, patient clinical features supporting antiphospholipid syndrome diagnosis, and repeating LA testing at least 12 weeks later).

In-depth review of the literature reveals many limitations of published data. First, most studies were performed on spiked samples. Studies with DOACs-treated patient samples are still needed, especially for inherited thrombophilia testing. Second, we did not address some specific technical issues, as for instance the potential different performance of activated charcoal (in terms of DOAC adsorption) according to the coagulation platform used or whether fresh or one freeze–thaw cycle samples were treated, since they were not specifically evaluated in any published study. Third, and as previously mentioned, many pre-analytical and analytical features with regard to thrombophilia testing in DOAC samples in vitro treated with activated charcoal differ between studies. It remains to be determined whether such differences may explain some of the discrepant results. Fourth, the type (commercialized or locally prepared) and the number of healthy volunteers from whom pooled normal plasma was prepared differ between the studies. Lack of standardization of the pooled normal plasma definition remains a limitation as no gold standard or universal international reference material is available.

## Conclusion

In the light of the present literature review, we provided 22 proposals for the reliable thrombophilia testing and accurate interpretation in samples from patients receiving DOACs using DOAC removal agents. This promising strategy is limited, for now, by the lack of data concerning inherited thrombophilia and the need of individual laboratory validation. This field is however in constant evolution with the development of easier-to-use packaging of DOAC removal agents such as DOAC Stop Liquid ^TM^ , and the development of tests that are less sensitive to DOACs. It is therefore likely that the results of future studies will clarify the role of DOAC removal agents and lead to updating the guidelines concerning their use. With the commercialization of new anticoagulant compounds (particularly FXI(a)/FXII(a) inhibitors) in the near future, healthcare professionals should also begin to unravel the interference of these compounds with the various thrombophilia assays and to assess the potential utility of activated charcoal-based products to circumvent any potential effect.
