# Supplementary material for: Usefulness and Limits of DOAC Removal Agents Based on Activated Charcoal in Thrombophilia Testing: Literature Review and Expert Proposals
Source: Thromb Haemost. 2025 Sep 29;126(9):867–82. doi: 10.1055/a-2695-2674 (PMC13521663; doi:10.1055/a-2695-2674)
Supplement: Supplementary file 1 [file 10-1055-a-2695-2674_29337008.pdf]

Supplementary Table S1 DOAC interference with activated protein C resistance testing.

Methods	Assay used	DOAC	DOAC concentration Median (range)	DOAC Removal agent	Impact of DOAC on APCR ratio Minimal C% without impact	Reference
Prothrombinase -based assays	***Pefakit APC-R Factor V Leiden***	Dabigatran	10-1000 ng/mL (spiking, pooled plasma)	None	Increase if ≥50 ng/mL	Lindhal et al.[18]
			167±97 ng/mL (ex vivo, n=3 FVL+/-)	None	Increase	Gessoni et al.[44]
			6-100 ng/mL (spiking, n=6 FVL-/- and 6 FVL+/-)	None	Increase If ≥6 ng/mL	Gessoni et al.[45]
			Not specified (ex vivo, n=12 FVL-/- and 6 FVL+/-)	None	Increase	Gessoni et al.[46]
			Not specified (ex vivo, n=30 patients)	DOAC-Stop	Increase	Favresse et al.[68]
		Rivaroxaban	10-1000 ng/mL (spiking, n=9 donors, n=1 FVL+/-))	None	Increase If ≥~800 ng/mL	Hillarp et al.[24]
			299±131 ng/mL (ex vivo, n=4 FVL+/-)	None	Decrease	Gessoni et al.[44]
			50-400 ng/mL (spiking, n=6 FVL-/-, 6 FVL+/- and 3 FVL+/+))	None	None	Gessoni et al.[46]
			Not specified (ex vivo, n=44 FVL-/- and 7 FVL+/-)	None	None	Gessoni et al.[46]
			Not specified (ex vivo, n=26 patients)	DOAC-Stop	None	Favresse et al.[68]
		Apixaban	Not specified (ex vivo, n=20 patients)	DOAC-Stop	Decrease	Favresse et al.[68]

Thrombosis and Haemostasis | DOI 10.1055/a-2695-2674 | © Thieme. All rights reserved.

					(not clinically relevant)	
		Edoxaban	100-500 ng/mL (spiking, pooled plasma)	None	Increase (≤100 ng/mL)	Douxfils et al.[35]
			10-742 ng/mL (spiking, n=10 donors)	None	None	Hillarp et al.[34]
			Not specified (ex vivo, n=10 patients)	DOAC-Stop	Increase	Favresse et al.[68]
APTT-based assays	***Coatest APC IL***	Dabigatran	10-500 ng/mL (spiking, pooled plasma)	None	Increase if ≥50 ng/mL	Kim et al.[23]
			10-1000 ng/mL (spiking, pooled plasma)	None	Increase if ≥50 ng/mL	Lindhal et al.[18]
			5000 ng/mL (spiking, pooled plasma)	DOAC Stop	Increase	Jacquemin et al.[57]
		Rivaroxaban	10-1000 ng/mL (spiking, n=9 donors, n=1 FVL+/-))	None	Increase If ≥~200 ng/mL	Hillarp et al.[24]
			~20-750 ng/mL (spiking, pooled plasma)	None	Increase If ≥140 ng/mL	Gosselin et al.[26]
		Apixaban	~20-750 ng/mL (spiking, pooled plasma)	None	Increase If ≥396 ng/mL	Gosselin et al.[26]
		Edoxaban	~20-480 ng/mL (spiking, pooled plasma)	None	Increase If ≥210 ng/mL	Gosselin et al.[26]
	HemosIL APC™ Resistance-V	Apixaban	86-447.3 ng/mL (spiking, pooled plasma))	None	None	Douxfils et al.[28]

APCR: Activated protein C resistance, DOAC: direct oral anticoagulant, FVL: Factor V Leiden

DOAC concentrations reported correspond to the range (min-max) of DOAC concentrations spiked or effectively measured in plasma samples prior to plasma treatment with DOAC removal agents. Concentrations are reported as median [IQR]. When available, DOAC concentration to which no interference is observed (≤) or from which interference has been reported (≥) are specified. DOAC impact on APCR measurement reported in the table results from the comparison between the measurement before and after DOAC addition in spiking experiment, before and after DOAC intake in ex vivo experiment or from the comparison between the measurement before and after plasma treatment with DOAC removal agent depending on the studies.

Thrombosis and Haemostasis | DOI 10.1055/a-2695-2674 | © Thieme. All rights reserved.
